# Parents’ perspectives on childhood antibiotic treatment in Ireland—a qualitative study

**DOI:** 10.1093/jacamr/dlaf176

**Published:** 2025-10-07

**Authors:** Anthony Maher, Eimear C Morrissey, Andrew W Murphy, Gerard J Molloy

**Affiliations:** School of Psychology, University of Galway, Galway, County Galway, Ireland; Centre for Health Research Methodology, School of Nursing and Midwifery, University of Galway, Galway, County Galway, Ireland; Institute for Clinical Trials, College of Medicine, Nursing and Health Sciences, University of Galway, Galway, County Galway, Ireland; Department of General Practice, College of Medicine, Nursing and Health Sciences, University of Galway, Galway, County Galway, Ireland; HRB Primary Care Clinical Trials Network, University of Galway, Galway, County Galway, Ireland; School of Psychology, University of Galway, Galway, County Galway, Ireland

## Abstract

**Background and objectives:**

Paediatric respiratory tract infections can be a common reason for antibiotic prescribing in primary healthcare. Despite stewardship efforts, prescribing patterns often diverge from evidence-based guidelines. There are limited explorations of how parental beliefs and behaviours shape clinical decision-making. This qualitative study explored parental perspectives on antibiotic treatment-seeking behaviour in Ireland.

**Methods:**

We carried out semi-structured interviews with 20 parents who had children under 8 years old in Ireland. The interviews were guided by the COM-B (Capability, Opportunity, Motivation – Behaviour) model. The interview data was analysed inductively, using reflexive thematic analysis. Following this, relevant themes and subthemes were mapped to the domains of the COM-B.

**Results:**

The study identified three key themes: (i) experiencing perceived knowledge gaps in antimicrobial resistance (AMR) and antibiotic use captured how participants described negotiating AMR as a personal health risk while also experiencing AMR as a distant policy; (ii) navigating professional gatekeepers described the role of consulting with the general practitioner (GP), the out-of-hours doctor paradox, trusting the pharmacist and seeing receptionists as hidden gatekeepers who all shaped access to care; and (iii) deciding when to act reflected how people sought pragmatic reassurance and managed illness escalation anxiety in making decisions about seeking treatment.

**Conclusions:**

The study underscores the need for socio-culturally tailored antimicrobial resistance messaging and interventions that address both parental concerns and systemic barriers. By centring parental voices, this research highlights opportunities to strengthen antimicrobial stewardship through improved communication, recognition and expanded roles for the primary healthcare team.

## Introduction

Antimicrobial resistance (AMR) is a global health threat, driven, in part, by the unnecessary use of antibiotics.^1^ Primary care is the setting where most antibiotic prescribing occurs, particularly in high-income countries, and paediatric respiratory tract infections (RTIs) remain among the most frequent reasons for these prescriptions.^2–5^ International guidelines emphasize the importance of antimicrobial stewardship (AMS) in reducing unnecessary prescribing, particularly for self-limiting infections such as viral RTIs.^5,6^ Yet, across healthcare systems, children receive antibiotics for RTIs in ways that can diverge from evidence-based recommendations.^7,8^ Although strategies have been implemented to reduce unnecessary prescribing, systematic reviews have noted that few studies have used qualitative methods to examine how parental beliefs and behaviours are managed during clinical consultations.^9,10^

This gap is particularly underexplored in the Irish context, where antibiotic prescribing rates remain high by European standards and where consultation dynamics may be shaped by unique structural and socio-cultural factors.^3,11^ Access to community healthcare in the Republic of Ireland is shaped by a mixed public–private funding model, where patients typically access general practitioners (GPs) for consultations in a continuity-based model. Patients may also access out-of-hours GP services that operate during evenings and weekends and are typically staffed by sessional doctors that do not typically involve ongoing patient–doctor relationships. In 2024, approximately 42% of the population qualified for free GP visits through the General Medical Services (GMS) scheme or the Doctor Visit Card (DVC) system.^12^ The GMS offers GP consultations without charge, including medications, for those on lower incomes or with specific health needs, while the DVC offers GP consultations without charge but excludes prescriptions. Since 2015, the roll-out of free GP care to all children under the age of 8 has marked a shift towards more universal healthcare access.^13^ However, this shift raises important questions about how such structural changes influence antibiotic prescribing patterns for children, particularly whether greater access leads to more frequent consultations and, potentially, increased antibiotic use.

Globally, existing literature suggests that clinicians may feel pressure from patients, parents and caregivers to prescribe antibiotics, even when clinical guidelines do not support such use.^14–16^ Additionally, UK studies show that clinicians often view prescribing for children as carrying higher perceived risks if antibiotics are withheld, adding an additional layer of complexity to these interactions.^17–19^ This tension could influence clinicians to balance AMS principles with the immediate demands of the consultation.^20^ However, much of the current evidence base around antibiotic consumption relies on quantitative methods, which potentially mask the socio-cultural and systemic factors that qualitative studies could identify as important.^21,22^ These approaches can emphasize quantitatively measurable variables and may not fully capture the complex, relational dynamics that shape antibiotic decision-making in everyday clinical encounters.^23–26^ Comparatively fewer studies have examined parents’ own perspectives, particularly in relation to how they conceptualize illness severity, engage with healthcare systems and understand the risks of antibiotic misuse.^27–29^ Where parental views have been explored, they have typically been framed around knowledge deficits or irrational beliefs.^30,31^ As a result, existing studies offer limited insight into the broader socio-cultural and relational factors influencing parental decision-making.

This study aimed to explore how parents of children under 8 in Ireland understand, navigate and potentially influence decisions around antibiotic treatment-seeking behaviour (ATSB) for RTIs within primary care. The study also considered how socio-cultural norms, informal advice networks and healthcare system factors may contribute to a heightened sense of urgency regarding ATSB. The study was guided by the Capability, Opportunity, Motivation – Behaviour (COM-B) model, which informed both the design of the interview schedule and the interpretation of findings.^32^ By mapping themes to COM-B, the study aimed to generate insights that could inform the design of targeted, behaviourally informed interventions to support AMS in primary care.

## Methods

### Study design and theoretical framework

A cross-sectional qualitative study, using semi-structured interviews, was employed. A qualitative descriptive approach was used, as it allowed the production of a ‘data-near’ and unadorned description of the experiences of participants.^33,34^ It is reported according to COREQ reporting guidelines (Table S1, available as Supplementary data at *JAC-AMR* Online).^35^

### Recruitment and sampling

Participants were recruited using purposive and snowball sampling strategies, with a maximum variation approach to capture a diverse range of experiences across parent age, gender and medical card status. This aligned with the inclusion criteria of being a parent or caregiver of at least one child under 8 years old, reflecting both the free GP care eligibility age and the relevant policy context. Recruitment materials were circulated from November 2022 to February 2023 across social media platforms (Twitter, LinkedIn, Facebook and Instagram) and on popular Irish parenting websites and forums (Mumsnet), Childminders Ireland and North Dublin Mums). Physical advertisements were distributed to general practices and community pharmacies in selected regions of Ireland, including counties Cork, Tipperary, Offaly, Kildare and Dublin, where healthcare professionals (HCPs) were asked to display them on noticeboards. Given the focused aim, and prior research showing insight from samples of this size, a target of 20 participants was considered appropriate.^36–38^ The concept of information power was used to guide decisions about sample adequacy during data collection.^39^

### Data collection

Interviews were conducted remotely via MS Teams between January and March 2025, lasting approximately 30–45 min. Interviews were audio-recorded with consent, and transcripts were automatically generated using MS Teams’ transcription function. The researcher manually checked all transcripts against the original audio files to ensure accuracy. Transcripts were returned to participants for verification, and participants were invited to clarify or elaborate on their responses.

The interview guide was structured around the COM-B model, with questions addressing capability (e.g. knowledge and understanding of antibiotic use), opportunity (e.g. access to healthcare providers) and motivation (e.g. perceptions of necessity and outcomes) asked. The guide was co-developed by the lead researcher and the wider academic team and reviewed by a public and patient involvement (PPI) panel consisting of three parents. Each PPI member also advised on recruitment approaches and provided feedback on data interpretation.

### Data analysis

Data were managed using NVivo12^®^ software by AM and ECM. The six-phase process of reflexive thematic analysis (RTA) was followed: (i) familiarization with the data, (ii) generating initial codes, (iii) searching for themes, (iv) reviewing themes, (v) defining and naming themes and (vi) producing the report.^40,41^ An inductive coding approach was applied, with themes identified from the semantic and latent content of the transcripts.^42^ Relevant themes and subthemes were mapped to the domains of the COM-B.

### Positionality

The interviewer was a male-registered pharmacist in Ireland and PhD candidate. Although there was no prior relationship with participants, the researcher approached the interviews with a degree of professional insight and presumptions based on community and hospital clinical experience. Reflexivity was embedded throughout the research process to account for this positionality, through a reflexive journal.

### Ethical considerations

Ethical approval was granted by the Research Ethics Committee at the University of Galway [2024.10.011].

## Results

A total of 20 parents participated (see Table 1). Three key themes were identified in the analysis, each comprising related subthemes (see Table 2).

**Table 1.** Participant characteristics

Pseudonym	Age	Gender	Number of children	Geographical location	Educational attainment	Healthcare access
Sinéad	31–40	Female	2	Rural	Completed higher education	Private healthcare
Seán	18–30	Male	1	Urban	Some higher education	State funded
Ciara	41–50	Female	3	Suburban	Postgraduate degree	Mixed
Niamh	31–40	Female	2	Rural	Completed higher education	Private healthcare
Orla	18–30	Female	2	Urban	Completed higher education	Private healthcare
John	41–50	Male	2	Suburban	Completed higher education	State funded
Hugh	31–40	Male	More than 3	Rural	Attended secondary school	Mixed
Áine	41–50	Female	2	Urban	Completed higher education	Private healthcare
Róisín	18–30	Female	1	Rural	Some higher education	Public healthcare
Patrick	31–40	Male	3	Suburban	Postgraduate degree	Private healthcare
Ciarán	18–30	Male	2	Urban	Attended secondary school	Mixed
Claire	Older	Female	More than 3	Rural	Some higher education	State funded
Aisling	41–50	Female	2	Suburban	Completed higher education	Private healthcare
Alan	31–40	Male	1	Urban	Postgraduate degree	Private healthcare
Nora	18–30	Female	2	Rural	Some higher education	State funded
Breda	31–40	Female	2	Suburban	Completed higher education	Private healthcare
Jack	41–50	Male	3	Urban	Attended secondary school	Mixed
David	18–30	Male	1	Rural	Some higher education	State funded
Una	31–40	Female	2	Suburban	Completed higher education	Private healthcare
Mary	Older	Female	More than 3	Rural	Attended secondary school	Private healthcare

**Table 2.** Themes and subthemes

Theme/subtheme	Description	COM-B alignment
**Theme 1: experiencing perceived knowledge gaps in AMR and antibiotic use**	Gaps in parents’ understanding around antibiotics and AMR	Psychological capability
*Subtheme 1.1: negotiating AMR as a personal health risk*	*Parents weighed the relevance of AMR to their own child’s health, often struggling to see its immediate impact*	*Psychological capability and reflective motivation*
*Subtheme 1.2: experiencing AMR as a distant policy*	*AMR was understood more as a societal or institutional concern than a personal or family-level issue*	*Psychological capability*
**Theme 2: navigating professional gatekeepers**	Parents navigating the roles of different healthcare actors, including who they trust, and how that trust intersects with expectations of responsibility	Social opportunity
*Subtheme 2.1: consulting with the GP*	*GPs were seen as the most trusted and authoritative source for antibiotic decisions*	*Social opportunity and reflective motivation*
*Subtheme 2.2 the out-of-hours doctor paradox*	*Parents valued quick access but were uncertain about the consistency or depth of care from unfamiliar doctors*	*Social opportunity and automatic motivation*
*Subtheme 2.3: trusting the pharmacist*	*Pharmacists were appreciated for their accessibility and advice, but rarely seen as primary decision-makers for antibiotics*	*Social opportunity*
*Subtheme 2.4: receptionists as hidden gatekeepers*	*Receptionists influenced access to care, sometimes creating frustration around appointment availability and perceived barriers*	*Physical opportunity and social opportunity*
**Theme 3: deciding when to act**	Parents expecting clarity and quick action but often encounter clinical uncertainty, especially in delayed or non-treatment strategies	Reflective and automatic motivation
*Subtheme 3.1: pragmatic reassurance*	*Parents sought antibiotics as a practical way to feel reassured and in control, even if unsure they were necessary*	*Reflective motivation*
*Subtheme 3.2: illness escalation anxiety*	*Worry about symptoms worsening pushed parents to act early and seek antibiotics as a precaution*	*Automatic motivation*

### Theme 1: experiencing perceived knowledge gaps in AMR and antibiotic use

Parents described a partial and, at times, conflicted understanding of antibiotics and their appropriate use. While there was widespread awareness that antibiotics do not treat viruses, this knowledge was not always consistent. Some parents expressed certainty in recognizing the biomedical limitations of antibiotics, while others acknowledged gaps among their peers or in their own understanding.

*I think most of my friends…don’t know the difference between bacterial and viral.* [Una]

Antibiotics were often viewed as a benchmark for the seriousness of illness. Parents’ attitudes ranged from pride in avoiding antibiotics to frustration when they were withheld, illustrating how different parents expressed diverse and sometimes contradictory expectations around antibiotic use.

*Some people… feel like the antibiotic is the only thing that’s going to make them better.* [Hugh]

#### Subtheme 1.1: negotiating AMR as a personal health risk

Parents described how external pressures impacted their healthcare-seeking decision-making, such as childcare exclusions and workplace obligations. In some cases, the practical benefits of receiving antibiotics outweighed concerns about resistance or side effects.

*In our old childcare setting… if a child went on an antibiotic, they were allowed to return to childcare after 48 hours.* [Áine]

The lack of alignment between clinical advice and parental expectations enabled allowed parents to rationalize partial adherence (e.g. not finishing prescription courses) without personal guilt. Despite an understanding of antibiotic adherence, parents admitted to starting treatment intermittently, particularly if symptoms improved, as obtaining the prescription itself was often seen as the most important step.

*We only gave it for like 3 days… we just gave up, because [my child] was fully better.* [Paul]

#### Subtheme 1.2: experiencing AMR as a distant policy

Not receiving an antibiotic was rarely perceived as a viable outcome, reinforcing the idea that obtaining a prescription marked the success of the consultation. For some, AMR was largely perceived as distant from personal antibiotic use. Within the sample, the concept of AMR was more closely associated with farming or animal products.

*Yeah, but that’s from animals… you don’t hear about it in a medical setting.* [Aisling]

Some participants drew comparisons with antibiotic access in other countries, highlighting how international norms could complicate domestic messaging and use about appropriate use. The restricted availability of antibiotics in Ireland over-the-counter seemed at odds with anecdotally looser policies abroad, subtly undermining the legitimacy of local stewardship efforts.

*…you just you walk in abroad, you don't need a prescription… and there's that side of it… why is it in other countries so readily available.* [Sinéad]

### Theme 2: navigating professional gatekeepers

For many parents, navigation of the healthcare system meant encountering a series of ‘gatekeepers’, who held varying degrees of authority and perceived trustworthiness. These professionals, while often respected, were also sources of frustration and uncertainty, shaping how and when parents accessed care.

#### Subtheme 2.1: consulting with the GP

As the central figure, trust was conditional, with parents deferring to GPs, but frustrated by perceived arbitrariness in prescribing decisions. Instances occurred where antibiotics were prescribed ‘just in case’, despite viral diagnoses. This underscored systemic inconsistencies in prescribing and diluted trust in healthcare guidance.

*They’re like ‘It’s a virus, we’re nearly sure it’s a virus, but we’ll give you these just in case.’* [Claire]

Reverence for GPs was paired with compliance within the consultations, even when treatment expectations were not met. While few parents were willing to challenge a GP’s decision outright, some reported feeling disappointed. Beneath this deference, however, frustration built up, particularly when parents felt their concerns were not taken seriously or when access appeared arbitrary. Inevitably, when clinical judgement clashed with strong parental conviction, strongly held beliefs about treatment efficacy could prompt assertive negotiation within the consultation.

*I really had to fight [the GP] to get that antibiotic.* [Nora]

Such actions reflect a quiet erosion of confidence, hinting at broader concerns about the credibility, consistency and fairness of antibiotic decision-making across the healthcare system.

#### Subtheme 2.2: the out-of-hours doctor paradox

Out-of-hours services were valued for their accessibility, especially when perceived urgent situations warranted antibiotics. Parents appreciated the walk-in nature of some of these services but feared the lack of depth and continuity an unfamiliar provider could provide. In this context, antibiotics (as the currency of care) often felt like the only meaningful offering out-of-hours services could provide.

*Like an out-of-hours doctor… they tend to give you antibiotics. Because, I suppose, they don’t know you.* [Orla]

Emotional cost, such as long waits, and impersonal care were common complaints, particularly among parents managing distressed or sick children late at night.

#### Subtheme 2.3: trusting the pharmacist

While parents valued pharmacists’ advice on logistical queries, they drew a firm boundary around diagnostic authority, especially for ambiguous or serious symptoms. Pharmacists’ scope was perceived as limited, especially for young children, and firmly secondary to doctors when it came to decision-making.

*Not that I don’t trust pharmacists… especially for a baby, I would prefer to have a doctor have done like a full examination.* [Ciarán]

#### Subtheme 2.4: receptionists as hidden gatekeepers

Receptionists were seen as powerful intermediaries; those who controlled access to care before the parent could consult the prescriber themselves. They were frequently described as obstructive or dismissive and grouped alongside the negative side of the GP discourse. These encounters appeared to add emotional and practical barriers to ATSB.

*Receptionists are just so difficult… they just don’t listen.* [Róisín]

*Participants appeared to view receptionists as an inevitable hurdle to access, and shared stories of being filtered or made to feel as though their concerns weren’t serious enough to warrant an appointment…*

*I think the receptionist thought they were a doctor themselves, like they’re ridiculous.* [David]

### Theme 3: deciding when to act

Parents’ decisions to consult a doctor were shaped by a careful weighing of symptom severity, cost, prior experience and emotional thresholds. While minor symptoms were often monitored at home, social influence or personal trial-and-error often prompted action.

*The first three or four times when they get sick, you see what sort of way they respond to it. And then after that…you get a better idea.* [Ciara]

#### Subtheme 3.1: pragmatic reassurance

In a stated ‘ideal world’, the primary reason for attending consultations was not to receive antibiotics, but to feel reassured through clinical assessment and clear guidance. Trust in the doctor’s assessment, and the legitimacy of being seen, helped parents accept a non-antibiotic outcome, even when initial expectations leaned towards a prescription.

*I know it’s sometimes just for my own peace of mind…because they’re checking for rashes, they check, listen to their chest and stuff like that.* [Breda]

This form of reassurance was illustrated as tactical, a way to ensure that something serious was not being missed. When decision-making responsibilities were redistributed from parents, confidence was instilled and the perceived urgency to seek antibiotics was anecdotally reduced.

#### Subtheme 3.2: illness escalation anxiety

Underlying many past accounts was a form of anxiety about deterioration, especially when symptoms persisted. This often built over days, creating a tipping point where parents felt they had waited long enough.

*If they’re not improving after kind of treating it for days …you’re kind of thinking that they probably would need something stronger.* [Alan]

A fear of parental inadequacy or ‘missing something’ intensified these feelings, particularly in cases where illness worsened suddenly or improved temporarily before a consultation. Parents shared stories of children seeming severely unwell at home, only to appear fine by the time they were seen, complicating how symptoms were presented and perceived. For some, repeated exposure to these cycles of uncertainty cemented the belief that only a clinical assessment (and often, antibiotics) could decisively resolve the issue.

*Every other time before that, I would say she got better when she had [antibiotics]… My gut instinct was right.* [Mary]

## Mapping to COM-B

Behavioural influences identified within conversations aligned with the core components of the COM-B model.

Psychological capability was demonstrated through varied understandings of antibiotic effectiveness and AMR. While many parents correctly identified that antibiotics do not treat viral infections, misconceptions persisted about their necessity for symptom relief, the distinction between bacterial and viral and the appropriate completion of antibiotic courses.

Reflective and automatic motivation were described in parents’ desire to reduce their child’s suffering and fulfil perceived social or caregiving responsibilities. Emotional drivers, including anxiety over symptom escalation and fear of being seen as neglectful, often accelerated decisions to seek care.

Physical and social opportunity influenced ATSB through structural and interpersonal factors. The availability of free GP care for children under 8 facilitated timely consultations, but short appointment windows were perceived to limit dialogue. Parents described how childcare policies, work obligations and healthcare gatekeepers (e.g. receptionists) shaped the feasibility of timely access. Pharmacists and out-of-hours services were seen as more accessible but variably trusted.

**Figure 1.**
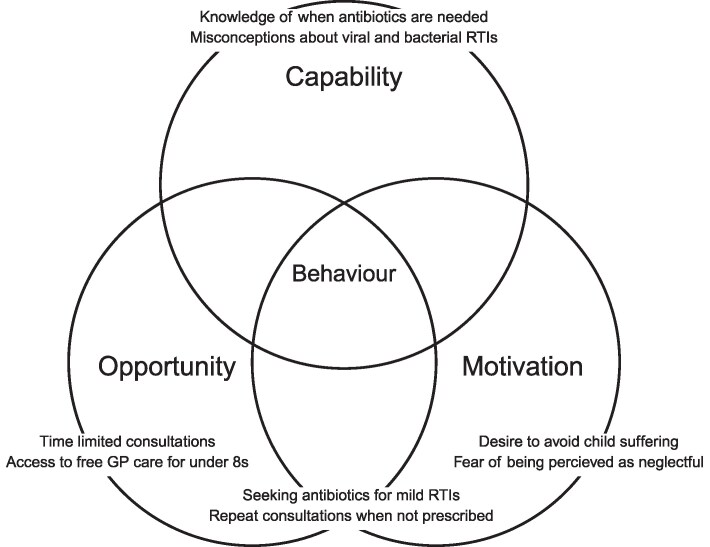
COM-B model map of identified qualitative findings.

Some behaviours are identified within intersections of the COM-B model domains (see Figure 1). The decision to re-consult was often driven by both motivational concerns (e.g. anxiety over symptom persistence) and opportunity structures (e.g. ease of accessing same-day appointments).

## Discussion

This study sought to explore parental understanding and how expectations and decision-making influences ATSB for childhood RTIs in Irish primary care. This was guided by an interest in how prior healthcare experiences and relational dynamics shape these behaviours.^43–46^ A key concept of navigating gatekeepers was described, with parents polarized between deference to and defiance of medical authority. This disconnect suggests that public health messaging around AMR may require culturally tailored reframing to resonate with parents.^47,48^ Moreover, it reflects a broader challenge faced globally: international norms around prudent antibiotic use may not have uniformly permeated local public consciousness.^49–51^ These findings underscore the challenges of translating stewardship principles into practice in a diverse set of socio-cultural contexts.

The intensity of parental advocacy for antibiotics, even in the absence of clinical justification, highlighted how socio-cultural narratives can override objective risk–benefit assessments.^52,53^ This reflects a broader trend in the literature, where parents’ trust is compromised when they perceive HCPs as overly rigid.^39,54^ Our findings align the triadic nature of paediatric consultations, involving the clinician (agent), parent (principal) and child (intermediary). Parents acted as proxies for their child’s wellbeing while navigating clinical authority.^55^ Distrust may also be compounded by inconsistent prescribing behaviour across clinicians, leading to confusion and suspicion around clinical decision-making.^56–58^ There is increasing recognition that recent changes in the polarization of political discourse in many contexts have led to a loss of trust in professional gatekeepers, particularly HCPs focused on global health issues in some contexts.^59^ In such consultation dynamics, some parents may adopt more assertive roles, including symptom exaggeration or pressuring for antibiotics, as a way to assert control over an opaque system.^38,60,61^

Participants appeared to demonstrate both conscious and unconscious gaps in their awareness of AMR. This aligns with prior UK and French studies, which found that AMR can be perceived as something individuals feel powerless to influence or as an abstract concern.^62,63^ This appears to reflect a form of conscious disengagement, where parents acknowledged AMR as a legitimate concern but deprioritized it in light of competing demands of everyday parenting.^49,64^ More often, however, perceived AMR knowledge gaps appeared unconscious; many parents either misunderstood the concept or had never had it clearly explained by a HCP. This is consistent with prior research, which identified public knowledge deficits in AMR, attributing them to weak or inconsistent health messaging.^65^

Parents also expressed a sense that HCPs were not always attuned to their emotional and practical needs. Clinicians, under time pressure, have been showcased in other contexts to deprioritize relational work in favour of clinical efficiency.^24,66^ Structural constraints in general practice may lead clinicians to shortcut explanations or avoid deeper dialogue, particularly around ‘low-risk’ cases.^60,67^ Receptionists were seen in this study as influential, though often invisible actors in the antibiotic-seeking process. This echoes prior findings of non-clinical staff playing a crucial, if informal, role in indirectly shaping clinical outcomes.^68–70^

While many AMS interventions have focused on improving knowledge or capability, our COM-B-aligned results suggest that both reflective and automatic motivation play a critical role. Several parents still equated the value of the consultation with a tangible outcome, often an antibiotic, reinforcing prior studies, which have found a transactional mindset in settings without universal access.^71,72^ Moreover, our findings align with the candidacy framework, illustrating how parents’ efforts to access antibiotics for their children are actively negotiated with HCPs who act as adjudicators of candidacy.^73^ These processes intersect with behavioural drivers described by COM-B, highlighting how parents’ knowledge, perceived risks and reassurance needs shape both their care-seeking behaviour and how candidacy is conferred or withheld in practice. This alignment could have implications for AMS strategies in publicly funded systems like Ireland’s, where mixed models of care may blur expectations around entitlement and outcome.^30,74^ Interventions that overlook motivational and opportunity-based influences, such as the emotional burden of caring for an unwell child, or the constraints of access, risk being effective.^75–78^ A COM-B-informed approach highlights the need to support not only clinical decision-making but also the relational and structural dynamics underpinning it.^79^

Our findings reinforce the need for clinicians to be attentive not only to symptoms but also to the underlying reassurance and safety-netting needs expressed by parents. Parents reported that clinicians appeared unaware of their need for emotional validation. This could speak to a persistent disconnect; while clinicians often view their role as clinical gatekeepers, the study highlights that parents seek validation and clarity from the encounter.^80,81^ Further, educational messages alone may fall short if they do not resonate with those without frequent clinical touchpoints. For receptionists, despite their role in the care-seeking journey, exclusion from AMR education or system-wide stewardship strategies is recognized, a latent oversight that could limit their potential as allies in AMS efforts.^82,83^ Pharmacists, by contrast, are increasingly positioned as accessible and trusted HCPs who could absorb some of the AMS functions currently carried by overburdened GPs, both in policy and in practice.^84–86^ In theory, community pharmacists in Ireland are well placed to offer triage, education and, in some scenarios, even non-prescription treatment pathways.^87,88^ However, our findings suggest that this potential remains largely unrealized. This perception aligns with broader findings that while pharmacists are trusted in their domain, they are not yet seen by the public as part of the clinical decision-making hierarchy.^89–91^

A strength of this study lies in the depth of the qualitative data collected. Through RTA, the study captures the complexity of parental beliefs, emotional reasoning and contextual constraints in ways that quantitative methods might not.^37,38^ The analysis benefitted from a diverse range of PPI input and parental voices. The integration of behaviour change frameworks further enhanced interpretive clarity, allowing findings to be mapped meaningfully for intervention design principles. These findings resonate with similar research, which suggests shared challenges across European healthcare contexts in aligning public behaviours with stewardship goals.^63,92^

However, several limitations exist. The sample may be subject to self-selection bias, with those who opted into the study potentially more health-literate to discuss antibiotic-related issues. As a result, parents with less confidence in health communication or limited engagement with health systems may be under-represented. The findings may also reflect experiences and decisions made within the primary care setting and may not generalize to emergency care contexts. While primary care represents the primary site of antibiotic prescribing in Ireland, the dynamics of urgency, accessibility and communication may differ significantly in other settings particularly LMIC settings, limiting broader applicability.^93,94^

This study highlights the complex landscape of parental ATSB in Irish primary care. To build on these findings, future research could explore how GPs and other prescribers perceive and respond to parental cues during clinical encounters. Additionally, tailored AMR interventions for parents, particularly those in high-prescribing communities, should be tested to ensure they reflect both behavioural science principles and local socio-cultural contexts. Recognizing broader socio-political undercurrents may help foster public engagement with AMR messaging and underscore the importance of framing stewardship efforts in ways that build trust rather than reinforce perceived hierarchies. AMS interventions could benefit from involving a broader range of HCPs, including pharmacists and nurses, whose roles in community-based prescribing and education appear underutilized.

## Supplementary Material

dlaf176_Supplementary_Data

## Data Availability

Recruitment materials, consent forms, and pseudonymized interview transcripts available on request.

## Supplementary data

Table S1 is available as Supplementary data at JAC-AMR Online.
